# Gut Microbiota and Host Metabolism: From Proof of Concept to Therapeutic Intervention

**DOI:** 10.3390/microorganisms9061302

**Published:** 2021-06-15

**Authors:** Patrice D. Cani, Emilie Moens de Hase, Matthias Van Hul

**Affiliations:** Metabolism and Nutrition Research Group, Louvain Drug Research Institute, Walloon Excellence in Life Sciences and BIOtechnology (WELBIO), UCLouvain, Université Catholique de Louvain, Av. E. Mounier, 73 B1.73.11, 1200 Brussels, Belgium; emilie.moens@uclouvain.be (E.M.d.H.); matthias.vanhul@uclouvain.be (M.V.H.)

**Keywords:** gut microbiota, obesity, metabolism, diabetes, correlation, causality, confounding factors, mice

## Abstract

The field of the gut microbiota is still a relatively young science area, yet many studies have already highlighted the translational potential of microbiome research in the context of human health and disease. However, like in many new fields, discoveries are occurring at a fast pace and have provided new hope for the development of novel clinical applications in many different medical conditions, not in the least in metabolic disorders. This rapid progress has left the field vulnerable to premature claims, misconceptions and criticism, both from within and outside the sector. Tackling these issues requires a broad collaborative effort within the research field and is only possible by acknowledging the difficulties and challenges that are faced and that are currently hindering clinical implementation. These issues include: the primarily descriptive nature of evidence, methodological concerns, disagreements in analysis techniques, lack of causality, and a rather limited molecular-based understanding of underlying mechanisms. In this review, we discuss various studies and models that helped identifying the microbiota as an attractive tool or target for developing various translational applications. We also discuss some of the limitations and try to clarify some common misconceptions that are still prevalent in the field.

## 1. Brief Introduction on the Gut Bacteria

The human gut is home to a variety of microorganisms, including bacteria, archaea, eukarya and viruses [1], collectively referred to as the gut microbiota. Most of the literature in this field relates to gut bacteria and is therefore the focus of this review. It has been estimated that the human gut harbors 10^13^ bacteria, a number that is similar to the number of human cells [2]. However, the (bacterial) gut microbiome, the gene pool encoded by the human gut bacteria, is composed of 100-fold more unique genes than the human genome [3]. Because of the coevolutionary history in harmony between microbes and their hosts and the intimate association between the gut microbiota and human health, this complex ecosystem has drawn increasing attention over the last decades [4].

Taxonomically, bacteria are classified according to phyla, classes, orders, families, genera and species. The human gastrointestinal (GI) tract is home to somewhere between 300 and 1000 different species that belong to only a few phyla. However, the gut microbiota is dominated by mainly two phyla: Firmicutes and Bacteroidetes, representing approximatively 85–90% of the total microbiota, whereas Actinobacteria, Proteobacteria and Verrucomicrobia are frequent, but generally minor constituents [5,6,7]. Interestingly, even though the gut microbiota composition of an individual is as unique as a fingerprint, the metabolic functions are remarkably conserved between individuals [8] and even between humans and other animal species [9]. Metagenomic analysis showed that 40% of the gut microbial gene pool is shared among all individuals around the world. This was termed the core microbiome [10]. However, despite the gaps in our knowledge, it is generally accepted that the gut microbes perform several key roles by breaking down different food components and nutrients and by synthetizing a range of metabolites. Some of these molecules exert primarily local activity, but others can be absorbed by the host and affect host health to different extents [11,12,13,14]. Among these, the most studied are the short-chain fatty acids, gases, vitamins and secondary bile acids. The importance of these gut microbiota-derived metabolites is reflected by the fact that they make up an estimated 10% of all molecules circulating in mammalian blood [15].

In this review, we discuss some of the difficulties and challenges the field of microbiome field still faces even after 10 years of substantial progress. We highlight different examples from the literature in which the role of the microbiota on metabolism is debated, and we also challenge the concept of establishing causality.

## 2. Correlation between Specific Gut Microbes and Health: Establishing Causality

**Figure 1:**
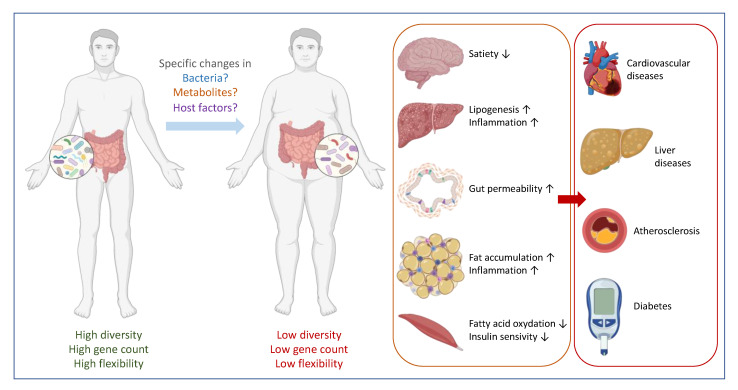
Gut microbes are linked with metabolic disorder. In a healthy situation, the composition of the gut microbiome is associated with a high diversity, high gene counts (high functionality) and a high capacity to adapt to potential stress conditions. An imbalance caused by specific changes in bacteria composition, metabolites production and/or host factors may result in a specific change in the microbiota with a state of low diversity and low gene counts, and poor flexibility. Poor flexibility means a lack of resilience or a difficulty to turn back to a basal and stable state. This contributes to an increased low-grade inflammation and organ malfunctions that in turn can lead to metabolic disorders, such as cardiometabolic conditions, liver diseases and (pre-)diabetes.

There is more and more evidence that the gut microbes influence not only tissues and organs in their close proximity, such as the intestinal cells and the liver, but also more distant sites such as adipose tissues, brain and muscles. This wide-ranging reach of the gut microbiota has been advanced in many studies, be it in humans or animals, as an explanation to link or even correlate specific changes in the microbiota composition with diseases. Indeed, it is not uncommon to read in the literature that a “disruption or imbalance of the homeostatic state of the gut microbiota” (also called dysbiosis) can be closely linked with health problems or specific diseases (Figure 1) [16,17]. This concept of dysbiosis is suffering from the lack of specificity and therefore cannot be used as a unique marker of disease or as unique risk factor. Moreover, how can one define a microbial imbalance if no one has defined what a ‘healthy gut microbiome’ is? Therefore, without a clear proof-of-concept of causality, the reported associations found in the literature are more than often purely and simply “mathematical” relations, meaning that the researchers are unjustifiably concluding that the higher (or lower) abundance of specific bacteria or a group of bacteria is the cause of a given disease. It is worth noting that a novel European initiative has been started with the aim to progress on these key questions, which is the “The International Human Microbiome Coordination and Support Action” which is aimed at providing international consensus on for instance clinical trial design, analytical standards, defining healthy microbiomes, as well as confounders, and eventually demonstrating causality in the field of microbiome (https://cordis.europa.eu/project/id/964590, accessed on 1 May 2021).

This binary view of ‘good’ versus ‘bad’ microbiota leads to a confusion. Indeed, the microbiota is so enormously lively and diverse that it is impossible to reduce it to black-and-white terms. Still, among the numerous studies that are published in the field of microbiota, many insist on labeling certain bacteria as either beneficial or harmful. While the vast majority of these publications are scientifically correct and relevant, there is a tendency to overinterpret correlations and sometimes even point toward causality when none are proven. This overenthusiastic reporting is undoubtedly a major reason why the field is still suffering from major criticism and has failed to convince certain specialists. There are many examples illustrating this persistent difficulty to be found in the exponentially growing microbiota-related literature (which, as of June 2021, had more than 10,000 articles published in 2020 alone, and a total of 42,800 since 1977, according to PubMed), not because the pioneering papers having reported these early observations were flawed, but because the techniques of analysis of the microbiome has since undergone considerable improvements, and yet, some researchers failed to abandon some “old” concepts that have been challenged since their discoveries. One of these examples is the paradigm of the increased “Firmicutes/Bacteroidetes ratio” which is frequently cited in the scientific literature as a hallmark of obesity. Initially discovered and proposed by Ley et al., this concept was very appealing at the moment of discovery because it englobed around 90% of the different taxa present in the gut and was very easy to understand [18]. Today, the validity of this ratio as a determinant of health status has been challenged due the great number of contradictory results reported in the literature [19]; many papers still continue to refer to it. The discrepancies might be explained by the lack of consideration of lifestyle-associated confounders known now to affect microbiota composition and/or diversity (see Section 3) and by the fact that these two phyla encompass many different bacteria, some of which are increased or decreased within the same phylum. For example, while it is true that several taxa belonging to the Firmicutes phylum such as *Clostridium, Lactobacillus* or *Ruminococcus* are often found to be increased in obesity, *Faecalibacterium prausnitzii*, another major member of the Firmicutes phylum, is one of the most abundant bacteria in the healthy human gut and is often found to be reduced in obesity and inflammatory bowel diseases [20,21]. In addition, the microbial community composition alone does not necessarily provide understanding of community function nor does it accurately predict the production of metabolites that may act on host metabolism. 

Another difficulty that the field of microbiome research faces is that, in light of the extreme complexity of the host-gut microbiota interrelationship, it is tempting to look for simple models or markers explaining and/or predicting certain biological responses. For example, Arumugam et al. suggested in 2011 that human gut microbiomes fall into distinct types or “enterotypes” based on three microbial patterns dominated by three specific genera: *Bacteroides*, *Prevotella* or *Ruminococcus* [22,23]. Later, this concept was extended with a fourth enterotype, the *Bacteroides* type 2 (Bact2) [24]. Interestingly, systemic inflammation levels and inflammatory bowel diseases are more prevalent in Bact2, thereby suggesting that the Bact2 enterotype might be indicative of an unhealthy microbiome constellation [24,25]. Although the classification of the human gut microbiome into separate enterotypes provides an attractive tool in the search for microbial markers related to certain diseases or specific host traits, the reality is more complex because we have to take into account the metabolic capacities of the gut microbes, the potential causes of such shift or apparent enterotype, what are the other microbes linked to a specific enterotype and, finally, whether a given enterotype is really causing a disease or increasing the risk of diseases. Moreover, it is important to note that, although advances have been made in identifying the individual microbial species that live in the human gut, a recent study estimates nearly 2000 species remain uncultured and therefore unknown [26]. This further highlights the gap of knowledge in this field and the difficulty to demonstrate the direct impact of specific microbes on human health [27,28]. Indeed, all these findings are not restricted to obesity since the gut microbiota composition has also been proposed to be playing a role on the severity of atherosclerosis, coronary artery disease and myocardial infarction [29,30].

Of note, besides these metabolic situations, a large number of other conditions (not discussed in this review) are linked to gut microbiota in the literature, although for the vast majority of them proof of causality is lacking, in humans at least. Among the numerous conditions showing correlations with some gut microbes, we can mention allergies, asthma, eczema, arthritis, stress, anxiety, neurodegenerative disorders, depression, autism and some cancers [31,32,33,34,35,36,37].

In addition, the lack of a comprehensive list of “beneficial” versus “deleterious” bacteria or even specific microbiota-based scores that would help us predict the risk of some diseases in a similar way as how we have been using certain specific biomarkers in the context of certain diseases has limited the implementation of microbiota-related research in the clinical arena. Such a list of “beneficial” versus “deleterious” bacteria currently remains impossible to provide if we want to remain at least scientifically correct. This has not stopped numerous companies from proposing commercial screening of fecal material in order to identify its composition. Some commercial players have even proposed doubtful interpretations of the microbiota composition by making claims of diseases risks. Researchers therefore have to keep a difficult balance between informing healthcare professionals about these naive and potentially hazardous malpractices and convincing them about the importance of gut microbiota in the maintenance of health.

Current efforts and pioneering studies that are presently under investigation with the aim to provide a more personalized approach could provide some relief in this matter by not only assessing the microbiome but by also studying and modeling the interactions with other key factors such as diet, genetics, environmental factors and exercise [21,27,28,38,39,40]. These pioneering studies may prove decisive for the future of microbiome research but are very expensive and slow to develop. 

Having said that, there are already some important markers that are widely accepted, even if they are still not causally linked with a specific disease. They are mostly used as indications that the gut microbiota composition and activity are deviating from a normal healthy situation. These markers are gene richness and microbiota diversity (richness is the total number of unique bacterial genes in the microbiome, while the diversity corresponds to the number of different species present in an individual). (Figure 1) [41,42].

## 3. The Major Confounding Factors

**Figure 2:**
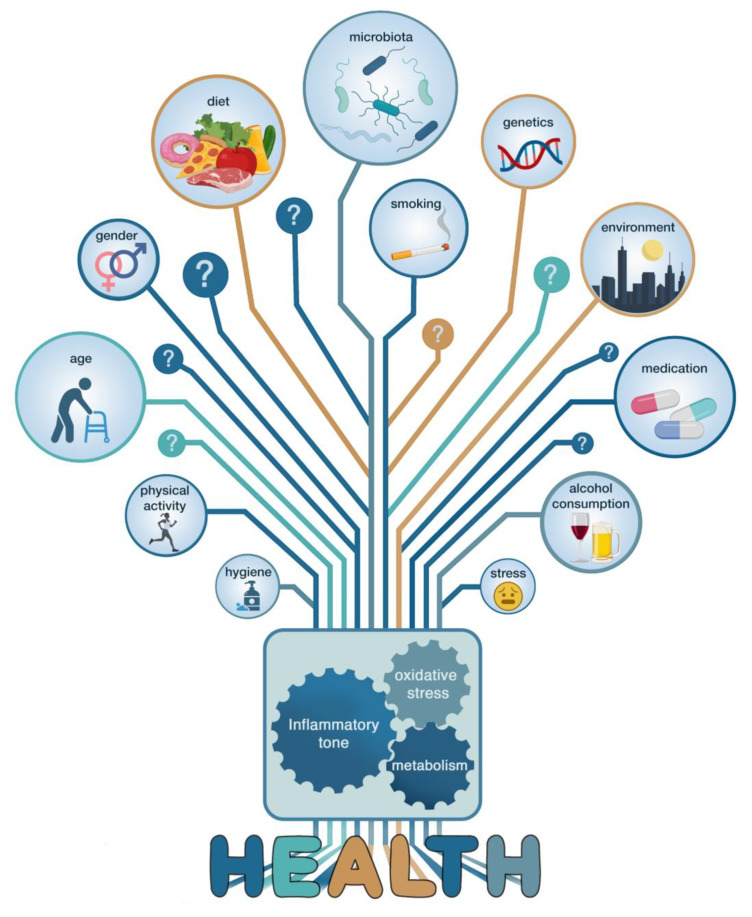
Many factors determine human health. Host characteristics and environmental factors interact and interfere with metabolic, inflammatory and oxidative stress pathways via direct and indirect mechanisms. Untangling these complex dynamics and relationship is one of the biggest challenges in understanding how human health is maintained.

It is widely accepted that the best evidence for cause-and-effect relations in health studies is provided by randomized, controlled and preferably blinded clinical experiments. These kinds of trials have resulted in linking different microbiota communities to a diverse range of human pathologies. These include not only immunological or metabolic disorders but also neoplastic, neurodegenerative and neuropsychiatric conditions. However, when associations are found between a certain health condition in humans and a particular gut microbiota composition/diversity, it is difficult to ascertain whether these associations are merely correlative or are a consequence of the health condition, or whether they might contribute to or even cause the health condition. Addressing this issue is challenging because of the intra- and inter-subject variability that exists, even among apparently healthy people. These differences are driven by complex combinations of environmental, physiological and lifestyle factors that exist between individuals (Figure 2). The identification of these confounders and effect modifiers is important but challenging. Not all confounders can be identified; some depend on subjective self-reporting, and others are very complex. Eliminating all of them is therefore impossible, and they may account for the many discrepancies observed in the literature between studies linking certain human conditions to divergent gut microbiota compositions.

The most obvious confounder is diet [39,43,44,45]. Both long-term dietary patterns and short-term interventions have been shown to induce changes in gut microbiota structure and function by providing nutrient sources and inducing environmental changes (shifts in pH, bile acids content, etc.) in the gut ecosystem [46,47,48], even if the precise mechanisms by which certain food components exert their influence on the gut microbiota composition remain uncertain. In addition, the gut microbiota is a direct target of certain food additives and contaminants [49]. Antibiotics are another evident source of gut microbiota disruption, but even drugs with no clear antibiotic activities can cause relevant modifications [50]. Five other covariates, i.e., gender, age, stool consistency (an indicator of bowel-movement quality), BMI and level of alcohol consumption have been identified as being among the strongest potential confounders in human cohorts from different regions including Belgium [51]), the Netherlands [52], China [53], the USA [27] and Japan [54] (Figure 2). Interestingly, a study from 2018 demonstrated that genetics and ethnicity had only a minor role in shaping the microbiota [55]. Consequently, the large role of the environment in forming the gut microbiome is also a major driver for the high diversity observed between populations from different regions [51]. Correctly accounting for such geographical variations is one of the challenges when reporting generalized microbiota-associated phenotypic variations [56].

Population-based variations and the many confounders question the universality of microbiome-based small-scaled research and make large-scale study design indispensable for characterizing microbiome shifts. Adding to the complexity, clinical trials/interventions in the field are faced with several challenges relating to sample preparation and processing, as there exists no standardization of methods for collection and analysis. Indeed, the results may be influenced by the method used to integrate data from different platforms, some inherent bias in primer selection during 16S ribosomal RNA sequencing and also a lack of consensus on how to collect the samples (e.g., fresh, frozen, with DNA/RNA stabilizer, or anaerobic conditions).

It is also important to note that, this review, like most of our own research and the majority of the studies in the field, focuses primarily on bacterial communities of the microbiome, even though it consists of many more members. Although this simplified approach is absolutely necessary to disentangle the complex interplay between host and microbial composition, it is very likely that this restricted view of the gut microbiota hampers our ability to fully understand the functioning and impact of the microbiome on the host biology. Interactions within and among microbial components are inevitable and undoubtedly of great importance. Combining inquiries aimed at fungal, viral and other microbial components will therefore be necessary in the future.

In order to identify true associations between disease and gut-microbiota composition, it is necessary to improve the characterization of the test subjects and to maximally measure the covariables, which may affect microbiota composition and interfere with the interpretation of the results. A good example of the progress that is being made in this regard is the recently published PREDICT-1 study [39], in which an impressive amount of profiling data was accumulated. However, despite their efforts, they are not exempt of the limitations inherent to data obtained using subjective self-reported measures. This study also highlights the current limitations due to the lack of ‘evidence-based microbiome’ studies [45].

## 4. What Are the Different Tools Used to Demonstrate “Causality”? 

### 4.1. Preclinical and Clinical Models “Combined”

In order to prove the causal link between specific bacteria and health or diseases, one of the gold-standard methodologies has been the use of germ-free (GF) mice. The ability to colonize these mice with predefined bacteria communities (gnotobiotic mice) or use them as recipients of fecal material from mice of interest or from human donors (humanized mice) has greatly accelerated progress in microbiome research. Of note, other mammalian models, such as nonhuman primates or pigs or nonmammalian models such as *Caenorhabditis elegans*, zebrafish and *Drosophila melanogaster* are not discussed in this review, although some of them may also serve as more ideal model systems than mice for simple microbiota and mechanistic aspects as reviewed by Leulier et al. [57].

In the context of metabolism, the pioneering works from Prof. Jeff Gordon in the early 2000s have been the first to show that GF mice were leaner than conventionalized mice [58] and later that GF mice were partially resistant to diet-induced obesity [59] or even that transferring the microbiota from obese human to GF mice induced higher fat mass gain than when the mice were colonized with the microbiota from lean subjects [60]. These studies undeniably showed the direct role of the overall microbiota on host metabolism. It is therefore not surprising that this model has become the most commonly used technique to investigate the role of the human or rodent microbiota on the onset of diseases. Indeed, the possible transplantation of gut microbiota of subjects or patients suffering from different conditions in recipient rodents and eventually the reproduction of the disease phenotype was clearly an important step to “prove” the causality. 

However, even if GF mice still represent a very useful tool for studying host–microbial crosstalk, over the years, concerns about the model have been raised as significant limitations to its use have emerged. For one, working with GF mice is laborious and demands skilled technicians that can follow protocols meticulously. However, beyond these practical issues, the mice develop important deficiencies associated with their confinement to a sterile environment. Indeed, the gut microbiota has been shown to be indispensable for proper immune, gut and brain development, which raises questions about the impact the recipient mice themselves have on the donor microbiota soon after the fecal microbiota transplantation (FMT) has been performed, despite a technically successful initial colonization. In addition, the use of germ-free mice is especially tricky when transferring microbiota from mice fed different diets (such as frequently used in obesity-inducing high-fat diet), because the composition of the gut microbiota is quickly changed by the diet of the recipient host [61,62]).

Hence, even with this model at hand, proving causality remains very difficult. To bypass the limitations of the GF models, depletion of the microbiota of fully developed mice using broad-spectrum antibiotics has been used for many years [63]. More recently, the procedure was improved by combining antibiotic treatments and bowel cleansing with polyethylene glycol (PEG) to better remove residual microbiota and prepare the mice for FMT. This represents a cheaper and easier solution that can be applied on any strain of rodents in a wider range of experiments [64,65]. However, the antibiotic-treatment does not allow for a complete depletion of the gut microbiota, and it is very difficult to control which strains survive and to what extent. Residual microbes might not always be a problem, but they may also create a niche that interacts with the donor microbiota. An additional problem is that many different protocols for the procedure circulate using different antibiotic combinations, doses and lengths of treatment. The lack of consensus on methodologies and standardizations is a recurrent problem within the field and is a potential source of biases [66]. 

The relevance of these kind of experiment for human–rodent studies has been questioned by some, due to the fact that the parameters that drove the dysbiosis in the first place (i.e., diet, lifestyle, disease phenotype, and human genotype) are no longer present in the recipient mice [67]. After microbiota transfer, some main taxa fail to colonize the recipient gut, and some others bloom in a nonrepresentative way, making it difficult to effectively reproduce the donor microbiota [68]. However, despite the differences between the donor and recipient mice, several studies showed that there is a remarkable proportion (±85%) of the human microbiota that can be successfully transferred into GF rodents [61], although others reports are challenging this number [64]. It is therefore important to acknowledge that many questions about these kinds of experiments remain unanswered: is the microbiota correctly transferred and mimicking that of the host donor? To what extent is the microbiota influenced by the host recipient mice, be it GF mice with an initially naïve and underdeveloped immune system or microbiota-depleted conventional mice? Is the genetic background of the mice influencing the response? All these questions are rightful and debated elsewhere in the recent paper by Walter et al. entitled “establishing or exaggerating causality for the gut microbiome: lessons from human microbiota-associated rodents” [69]. The authors pointed that many experiments lack rigor in their experimental designs and use inappropriate statistical analyses [69]. Even if these methods have their flaws and do not always sufficiently reflect the donor’s microbiome, researchers have been able to show that the microbiota, at least in part, contributes to the severity of disease. 

In a study using different experiments based on FMT methods from NAFLD patients to mice, researchers were able to identify *Klebsiella pneumoniae* as being the trigger of NAFLD in recipient mice [70]. In a similar manner, the transfer of microbiota from metformin-treated mice into germ-free mice resulted in an improvement of glucose metabolism, suggesting that metformin-altered microbiota is able to pass on beneficial effects from one host to the next [71]. Modulating the gut microbiota by administration of inulin-type fructans (a prebiotic) confers a health benefit in the context of obesity-associated metabolic disorders, even though the response to dietary fiber supplementation was dependent on the initial microbiome composition [65]. The long-term effects of bariatric surgery (Roux-en-Y gastric bypass or vertical-banded gastroplasty) on the gut microbiota were assessed by FMT in GF mice. These mice showed reduced fat deposition and lower respiratory quotient, without affecting food consumption, implying the causal role of gut microbiota in the reduction of adiposity observed after bariatric surgery [72]. 

Although large-scale trials on human-to-human fecal matter transplant are scarce and information on the subject relies heavily on case reports, some studies show that interfering with gut microbiota composition may causally affect certain disease states. Vrieze et al. investigated the beneficial effect of the infusion of gut microbiota from lean donors to patients suffering from metabolic syndrome on insulin sensitivity and glucose metabolism. They analyzed the gut microbiota composition and noted an increase in butyrate-producing bacteria (*Roseburia intestinalis* and *Anaerobutyricum soehngenii*, formerly designated as *Eubacterium hallii*), suggesting their potential implication in the improvement of insulin sensitivity [73]. In 2017, another study from the same group demonstrated the importance of baseline microbiota on the response to FMT in the improvement of insulin sensitivity (metabolic responders versus nonresponders) [74].

The potential of fecal transplantation on clinical outcomes of several disease states is illustrated by the many clinical trials that are currently in the pipeline, not only in the context of metabolism but also in many other pathological situations, such as in the context of neurological disorders. Clinical trials are organized in the treatment of severe and enduring anorexia nervosa, bipolar disorders, autism and Parkinson’s disease, among others. Other clinical trials recruit patients in the framework of multidrug resistance or for the therapy of intestine-associated disorders, such as ulcerative colitis, Crohn’s disease and also fecal incontinence. Many studies also focus on FMT in connection with metabolic syndrome, while a few studies are interested in different disorders, such as the link between microbiota and severe acute graft-versus-host disease or metastatic melanoma who failed immunotherapy (clinicaltrials.gov consulted on 17 May 2021). This shows the potential of gut microbiota modulation in the treatment of a large number of conditions. Although we have to acknowledge that in the case of *Clostridioides difficile* infections, FMT is no longer at the research stage but is a recommended therapy [75].

### 4.2. Using Specific Bacteria to Show Causality

As discussed earlier in this review, it is still very difficult to fully decipher the role of specific microorganisms or unique bacteria, especially within a complex community such as the gut microbiota. However, some species have drawn attention as they seem to have a greater impact on maintaining the stability of the microbial ecosystem than other species. Loss of these “keystone species” might have profound implications on the regulation of the overall community or at least on host metabolism. Among these, the commensal bacteria *Faecalibacterium prausnitzii* is probably the best known, although its protective role has mostly been described in the context of intestinal inflammation [76]. Given that the present review is primarily focusing on endocrine and metabolic functions, we abstain from describing all the bacteria that have been linked with diseases. It should be noted, however, that although many examples exist, many are still debated, including the role of specific bacteria on intestinal disorders and cancer (e.g., *Fusobacterium nucleatum*) (for review [77]). 

#### 4.2.1. The Case of *Subdoligranulum*

Recently, our attention was drawn to the *Subdoligranulum* genus, as this taxon fitted the criteria of potentially beneficial bacteria. For example, we found that metabolic improvements in prebiotic-treated obese and diabetic mice were associated with an increase in *Subdoligranulum* levels by almost 4-fold [78]). In humans, we found that obese patients with higher levels of *Subdoligranulum* had a better metabolic response after caloric restriction than obese patients with lower *Subdoligranulum* levels [41]. In addition, the anti-diabetic drugs metformin and acarbose increased fecal *Subdoligranulum* relative abundances [50,79]. Several studies also reported that *Subdoligranulum* genus correlated negatively with different parameters related to metabolic risk such as C-reactive protein (CRP), fatty liver index, homeostasis model assessment-insulin resistance (HOMA-IR), and glycated haemoglobin (HbA1c) and positively with HDL cholesterol [79,80,81]). Moreover, *Subdoligranulum variabile*, the only species of this genus isolated and described so far, was shown to produce butyrate [82]), a short-chain fatty acid to which health-promoting abilities are attributed [83]. However, despite all these observations, supplementation of *Subdoligranulum variabile* failed to exert any beneficial effects in a diet-induced obesity mouse model [84] and is not yet possible to be tested in humans. This study is a good example of why it is crucial to assess the validity of an associative observation between certain microbial components and health/disease conditions.

#### 4.2.2. The Case of *Akkermansia muciniphila*

*Akkermansia muciniphila* is an interesting example of the feasibility to move microbiome science from the bench to the bedside, even after only 10 years of research. Since its discovery in 2004, the interest generated by this bacterium has increased exponentially. There are now over 1300 papers on the subject, with more than 477 papers published in 2020 only. However, most of these studies did not investigate the direct impact of *Akkermansia* on the host but were confined to correlating its presence with different states of health. Nevertheless, enough evidence has now accumulated for *A. muciniphila* to be regarded by several scientists as a next-generation beneficial microbe [85,86,87]. 

Interest in *A. muciniphila* was first piqued by the observation that this bacterium was less abundant in the microbiota of obese mice, both in genetically obese/diabetic mice and in high-fat diet-fed mice [78,88]. In these mice, the abundance of *A. muciniphila* was inversely correlated with the body weight, adiposity, inflammation markers, insulin resistance and glucose intolerance. In addition, it was observed that prebiotic feeding (e.g., with inulin-type fructans) strongly increased the presence of *A. muciniphila* and that this effect was correlated with lower obesity and fat mass, improved insulin resistance, lower liver steatosis and gut permeability [78]. These promising observations led to further explorations into whether this bacterium might be of interest in humans as well, and not long after, decreased abundance of *A. muciniphila* was also reported in several human pathological conditions, such as obesity, type 2 diabetes, hypertension, hypercholesterolemia and liver disease (for review [89]). However, although data were consistent and ample, they remained purely correlative. In a logical next step, numerous studies adopted a proof-of-concept strategy by exploring the role of *A. muciniphila* using different animal models. In a nutshell, these combined studies demonstrated that the administration of *A. muciniphila* protected against several cardiometabolic features (i.e., lowers body weight and fat-mass gain, hepatic steatosis, inflammation, cholesterol levels and atherosclerosis, improves insulin sensitivity and restores gut barrier function) [88,90,91,92,93,94,95,96]. 

However, human translational evaluation remains the ultimate goal. Therefore, a study was recently set up to explore the feasibility and the safety of *A. muciniphila* in humans. The main objectives of this first exploratory study were on the one hand to evaluate the feasibility, safety and tolerance of *A. muciniphila* supplementation, either as live bacteria or in a pasteurized form, and on the other hand to explore the metabolic effects of *A. muciniphila* supplementation in humans. In a double-blind placebo-controlled randomized study, Depommier et al. investigated the impact of a daily administration of *A. muciniphila* for 3 months in adults suffering from prediabetes and metabolic syndrome (Microbes4U^®^ cohort) [87]. This exploratory proof-of-concept study demonstrated that daily administration of *A. muciniphila*, either alive or pasteurized, was safe and well-tolerated but also that the supplementation with *A. muciniphila* was limiting the worsening of cardiometabolic disorders associated with overweight and obesity (i.e., insulin resistance, liver inflammation, gut permeability, hypercholesterolemia and, to a lesser extent, fat mass and waist–hip ratio) [87]. Although the mechanisms of action are not yet fully elucidated, Depommier et al. suggested in a follow-up study that the beneficial effects of *A. muciniphila* could be linked to a specific increase in circulating 1-Palmitoyl-glycerol (1-PG) and 2-Palmitoyl-glycerol (2-PG). These bioactive lipids, belonging to the endocannabinoidome (eCBome), were identified as endogenous activators of peroxisome proliferator-activated receptor alpha, thereby contributing to a better health outcome [97].

#### 4.2.3. The Case of *Prevotella Copri*

*Prevotella copri* is a frequent, yet not ubiquitously present, inhabitant of the human gut [10]. *P. copri* has been associated with insulin resistance and glucose intolerance, due to its ability to augment circulating levels of branch-chain amino acids (BCAAs) [98] and has also been linked with inflammatory diseases [99,100]. Conversely, *P. copri* has also been linked to improved glucose and insulin tolerance via the production of succinate upon the fermentation of dietary fibers [101,102]. Although both conflicting observations were based on solid data from human studies and were confirmed in mouse interventional studies, these proof-of-concept studies did not allow one to draw a final conclusion. Whether the effects of *P. copri* could be dependent only on the dietary conditions is an interesting question that requires further investigation, since another recent study strongly linked this bacterium with better health [39]. 

## 5. Conclusions and Perspectives

**Figure 3:**
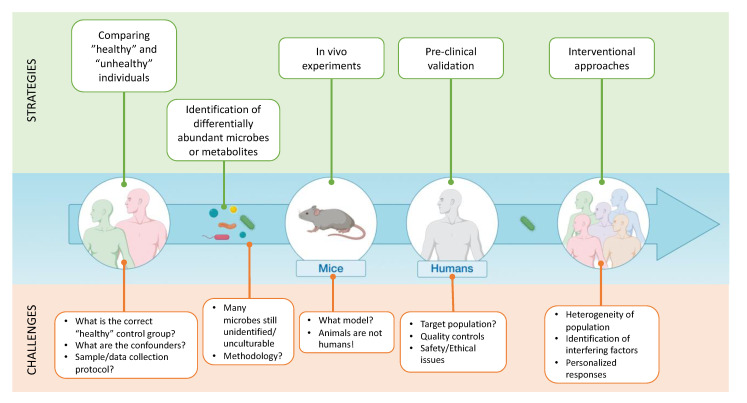
Challenges and strategies for establishing causality. Correlative evidence obtained from comparing healthy and diseased cohorts may identify potential beneficial bacteria or metabolites. Causality testing in animal models and humans are needed to validate the observational findings and can help decipher mechanistic or host-response effects. In very rare cases, this may lead to the development of new therapeutic approaches. Every step of this top-down strategy faces several intrinsic challenges and difficulties.

In the present review, we have discussed and challenged how and why the microbiome provides an opportunity, as well as a risk, for practitioners and consumers to be misinformed, especially when the evidence-based medicine is not yet fully realized. Indeed, the microbiota-related literature is relatively new and has experienced an exponential bloom. This upsurge in scientific interest has been sparked by a growing appreciation of the importance of the gut microbiota in the health of the host. The subsequent race to characterize divergent microbiota compositions in a large number of contexts has led to the discovery of many links between host nutrition, the gut microbiota and metabolic and immune functions (Figure 2). However, the initial excitement has clouded our ability to look beyond mere correlations and truly recognize and characterize how bacteria causally influence normal biology. Moreover, the recent advance in culturomics will lead to the discovery of a new genera or species potentially playing a major role on human health [26]. This also adds another layer of complexity since these new taxa may also change the vision or the conclusion of the past studies, having unintentionally ignored the presence of these taxa. One of the key examples is the recent discovery of *Dysosmobacter welbionis*, isolated from the human gut and present in 70% of the general population at a relatively high abundance (0 to 9%), inversely correlated with obesity and diabetes; this novel bacterium is able to reduce obesity, diabetes and inflammation in diet-induced obese mice [103]. Moreover, our propensity for simple and straightforward conclusions has led to erroneous narratives that have added more confusion in an already complicated field of study (Figure 3). Although the gut microbiota undoubtedly plays a significant role in host health, there are dangers in shifting responsibility for a wide range of illnesses to just certain gut microbes or microbial constellations. We must be careful not to easily invoke “a dysbiosis” as convenient scapegoat for the spread of certain diseases. New insights have shaken some dogmas and serve as a reminder that to researchers nothing can be sacred and everything should be challenged (Figure 3). Nevertheless, the research to date has sufficiently advanced to suggest that microbiota-based treatment could become useful in preventing and countering certain disorders. One thing that microbiome research has taught us so far is that taking care of our gut inhabitants is primordial for maintaining a healthy state. Therefore, understanding gut microbiota modifications and developing tools and strategies to steer microbial communities away from a state of dysbiosis and direct it toward a desirable healthy state are some of the priorities of researchers in the field. Achieving such changes is probably multifaceted. The quest for a single magic bullet is an illusion of the past. Prebiotics, probiotics, postbiotics and bacteriophages need to be combined with lifestyle changes to obtain significant results. However, bad habits are hard to break, and good habits hard to form. Raising awareness by communicating clear, but correct, messages, while avoiding sensational headlines is therefore a considerable part of the strategy to introduce microbiota-based treatment into mainstream clinical practice. 

Incredible progress has been achieved in only few years, and the numerous efforts have raised the field of the gut microbiome as an important contributor. However, we have to acknowledge that, as in more “classical” fields of investigations such as physiology and metabolism, many questions remain to be answered and numerous mechanistic experiments, randomized controlled trials, and interventional studies will be useful for obtaining the answers.
